# Omic analysis of the endangered Taxaceae species *Pseudotaxus chienii* revealed the differences in taxol biosynthesis pathway between *Pseudotaxus* and *Taxus yunnanensis* trees

**DOI:** 10.1186/s12870-021-02883-0

**Published:** 2021-02-19

**Authors:** Chunna Yu, Chengchao Zhang, Xinyun Xu, Jiefang Huang, Yueyue Chen, Xiujun Luo, Huizhong Wang, Chenjia Shen

**Affiliations:** 1 College of Life and Environmental Sciences, Hangzhou Normal University, Hangzhou, 310036 China; 2 Zhejiang Provincial Key Laboratory for Genetic Improvement and Quality Control of Medicinal Plants, Hangzhou Normal University, Hangzhou, 310036 China

**Keywords:** Metabolome, Taxoids, Taxol biosynthesis pathway, Transcriptome, Resource utilization

## Abstract

**Background:**

Taxol is an efficient anticancer drug accumulated in *Taxus* species. *Pseudotaxus chienii* is an important member of Taxaceae, however, the level of six taxoids in *P. chienii* is largely unknown.

**Results:**

High accumulation of 10-DAB, taxol, and 7-E-PTX suggested that *P. chienii* is a good taxol-yielding species for large-scale cultivation. By the omics approaches, a total of 3,387 metabolites and 61,146 unigenes were detected and annotated. Compared with a representative *Taxus* tree (*Taxus yunnanensis*), most of the differentially accumulated metabolites and differential expressed genes were assigned into 10 primary and secondary metabolism pathways. Comparative analyses revealed the variations in the precursors and intermediate products of taxol biosynthesis between *P. chienii* and *T. yunnanensis*. Taxusin-like metabolites highly accumulated in *P. chienii*, suggesting a wider value of *P. chienii* in pharmaceutical industry.

**Conclusions:**

In our study, the occurrence of taxoids in *P. chienii* was determined. The differential expression of key genes involved in the taxol biosynthesis pathway is the major cause of the differential accumulation of taxoids. Moreover, identification of a number of differentially expressed transcription factors provided more candidate regulators of taxol biosynthesis. Our study may help to reveal the differences between *Pseudotaxus* and *Taxus* trees, and promote resource utilization of the endangered and rarely studied *P. chienii*.

**Supplementary Information:**

The online version contains supplementary material available at 10.1186/s12870-021-02883-0.

## Background

Taxol (generic name paclitaxel) and its related products have been widely adopted and approved for the effective treatments of various types of cancers including breast, lung, non-small cell lung cancers [1]. For years, the barks and twigs of Taxaceae plants, *Taxus* trees in particular, are the major source of natural product taxol and its derivatives [2, 3]. As the market demand for taxol increases annually, the illegal logging continues [4]. Due to over-exploitation and human activities, the population size of Taxaceae is shrinking [5, 6].

Taxaceae is the smallest family of conifers, consisting of six genera, including two former genera of Cephalotaxaceae (*Amentotaxus* and *Cephalotaxus*) and four so called ‘core’ genera of Taxaceae (*Austrotaxu*s, *Pseudotaxus*, *Taxus* and *Torreya*) [6, 7]. Owing to the economic value of taxol, the study on Taxaceae trees has drawn numerous attentions in the past years [8].

Taxol is a classic representative of the more than 400 defined taxoids, which contain the unique taxane skeleton [9]. Not only taxol, but also some other taxoids have exhibited potent anti-tumor activities [10, 11]. To date, more and more compounds with the taxane core have been isolated and identified in various Taxaceae trees [12]. Four novel taxane derivatives with high anticancer potency were isolated from the ethanol extract of the whole plant of *T. wallichiana* [13]. Inhibitory effect of 13 taxane diterpenoids from *T. chinensis* on the proliferation of HeLa cervical cancer cells was evaluated and their chemical structures were deciphered [14]. Homoharringtonine, which is originally isolated from the genus *Cephalotaxus*, is another natural product in cancer chemotherapy [15, 16]. However, information on the occurrence of taxol and other taxoids in *P. chienii* is very limited.

The previous studies have revealed three representative groups of taxoids, including 13-hydroxylated taxoids (baccatin III, paclitaxel, etc.), 14-hydroxylated taxoids (taxuyunnanin C, yunnaxan, etc.), and 11(15 → 1)-*abeo*-taxoids [17]. As one of the most important 13-hydroxylated taxoids, Taxol’s specific synthetic branch involves 19 steps from the diterpenoid progenitor geranylgeranyl diphosphate (GGPP), which is derived via the plastidial 2-C-methyl-D-erythritol phosphate (MEP) pathway, to taxol [9]. To form the taxane skeleton, cyclization of GGPP to taxa-4(5),11(12)-diene is catalyzed by a slow-starter enzyme, taxadiene synthase (TS) [18]. Then, multiple modification enzymes, particularly from the CYP450, acyltransferase, aroyltransferase and benzoyltransferase families, participate in the structural modification of the skeleton [19–21]. A series of CYP450s, including the 2α-, 5α-, 7β-, 9α-, 10β-, and 13α-hydroxylases, are involved in the oxygenation steps of taxadiene at the C^− 2^, C-5, C-7, C-9, C-10, and C-13 positions [22]. Meanwhile, several other transferases are considered to be involved in taxol biosynthesis, such as taxadien-5α-ol O-acetyl transferase (TAT), taxane-2α-O-benzoyltransferase (TBT), 10-deacetylbaccatin III-10-O-acetyltransferase (DBAT), baccatin III-3-amino, 3-phenylpropanoyltransferase (BAPT), and 3′-*N*-debenzoyl-2′-deoxytaxol-*N*-benzoyl transferase (DBTNBT) [23].

The taxol biosynthesis pathway is not a linear route but rather a complex network consisting of various divergent routes. For example, CYP450-mediated oxygenation steps engender a number of taxusin-like intermediate metabolites, such as 2α-hydroxytaxusin and 7β-hydroxytaxusin [22]. As a 14-hydroxylated taxoid, Taxuyunnanin C, has a significant effect on the enhancement of neuron growth factor activity, in cell suspension cultures of *Taxus* species [24, 25]. A 14β-hydroxylase (T14OH) is involved in the diversion of the pathway towards 14β-hydroxylated taxoids, such as taxuyunnanin C [26].

*T. yunnanensis*, an endangered and slow-growing tree, is mainly distributed in Yunnan Province of China [27]. Phytochemical studies have isolated and identified many taxoids and other chemical compounds with biological activities from the seeds and twigs of *T. yunnanensis*. For examples, three novel taxane diterpenoids, namely baccatin VIII, baccatin IX, and baccatin X, were isolated from an ethanolic extract of the twigs and leaves of *T. yunnanensis* [28]. A novel heteropolysaccharide (TMP70W) with in vitro antitumor activity was structural identified from *T. yunnanensis* [29]. *P. chienii*, the sole species in the monotypic genus of *Pseudotaxus*, is another endangered yew and mainly distributed in the South China [5]. Morphological analysis showed a high similarity between *P. chienii* and the species from *Taxus*, differing from each other notably in the color of aril: white in *Pseudotaxus* and red in *Taxus* [6]. However, the differential accumulations of taxoids between *Pseudotaxus* and *Taxus* are largely unknown.

Technical advances in large-scale identification of genes and metabolites have facilitated to investigate the variations between two close species [30, 31]. Recently, several transcriptomes and metabolomes of *T. yunnanensis* have been reported [32–34]. So far no omic data of *P. chienii* was available. In the present study, an integrated metabolomic and transcriptomic approach was employed to elucidate the differential accumulations of taxoids between *P. chienii* and *T. yunnanensis* and the underlying mechanisms. Our study may provide valuable information for the conservation and comprehensive utilization of these two endangered Taxaceae species.

## Results

### Quantitative analysis determined the occurrence of taxol in *P. chienii* and the variations in taxoids between *P. chienii* and *T. yunnanensis*

**Fig. 1:**
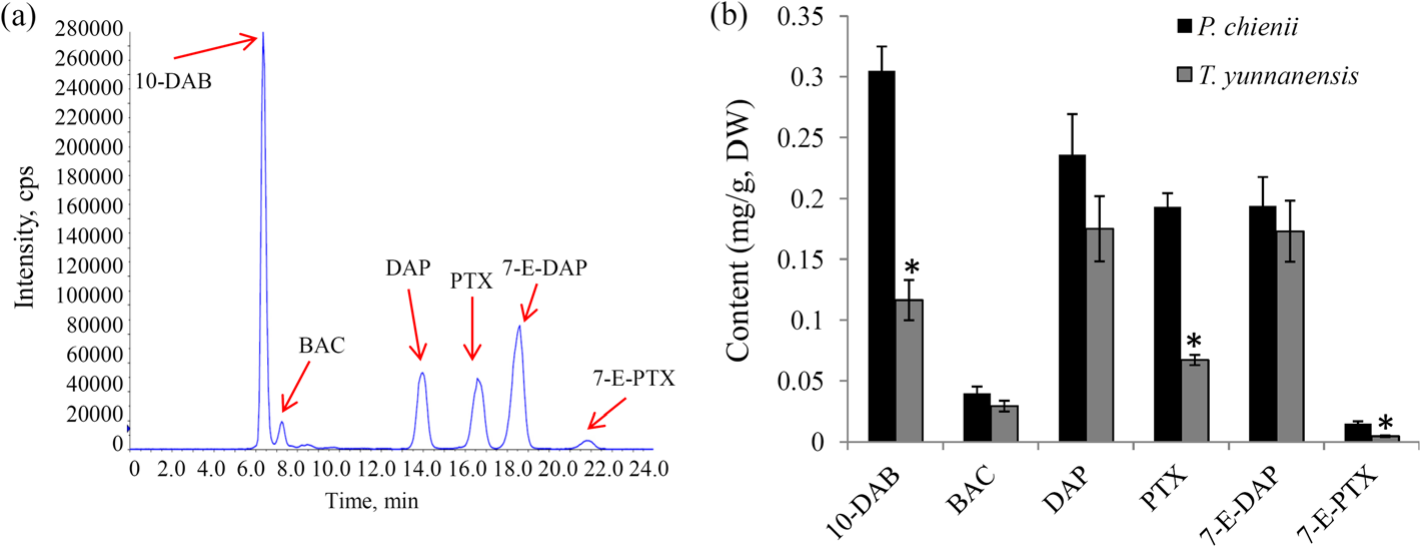
Determination of the contents of taxoids in *P. chienii* and *T. yunnanensis*. **a** A representative TIC chromatogram of six taxoids. 10-DAB: 10-deacetylbaccatin III; BAC: baccatin III; DAP: 10-deacetylpaclitaxel; PTX: paclitaxel; 7-E-DAP: 7-epi 10-desacetyl paclitaxel; 7-E-PTX: 7-epipaclitaxel. **b** The contents of six taxoids in *P. chienii* and *T. yunnanensis*. A *P* value < 0.01 was considered to be statistically significant and indicated by ‘*’

To date, the occurrence of taxol in *P. chienii* is unknown. Herein, a target LC-MS approach was applied to check the occurrence of taxol and other taxoids, such as 7-E-DAP, 10-DAB, BAC, DAP, and 7-E-PTX, in *P. chienii*. Our data showed that all selected taxoids were detected, confirming the occurrence of taxoids in *P. chienii* (Fig. 1a). Quantitative analysis revealed the variations in taxoid contents between *P. chienii* and *T. yunnanensis*. In particular, 10-DAB, PTX, and 7-E-PTX highly accumulated in *P. chienii*. No significant differences in the contents of BAC, DAP, and 7-E-DAP were obeserved between *P. chienii* and *T. yunnanensis* (Fig. 1b).

### Overview of the metabolites

**Fig. 2:**
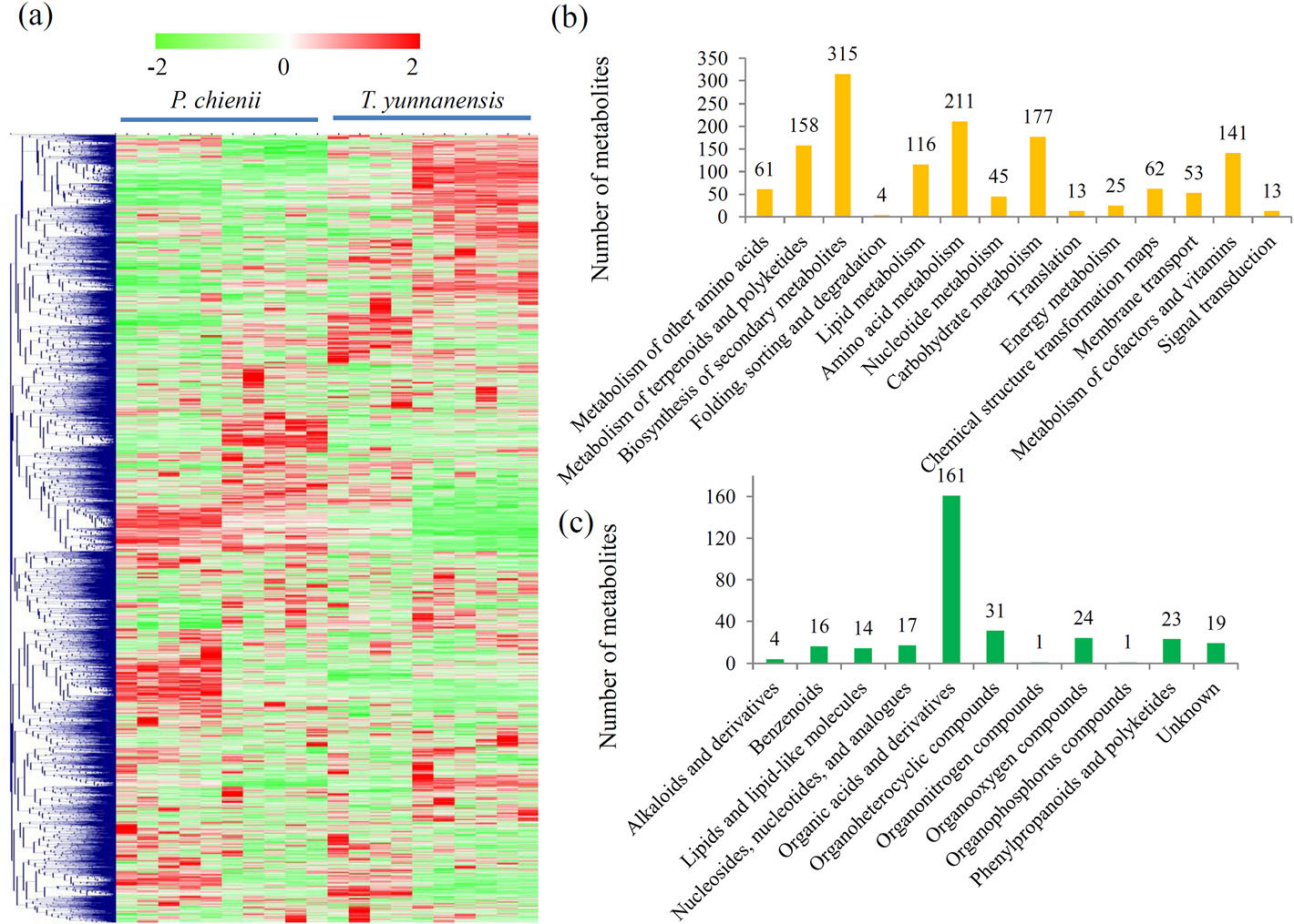
The variations in the abundance of metabolites between *P. chienii* and *T. yunnanensis*. **a** A heatmap of the metabolites identified in the metabolomes of *P. chienii* and *T. yunnanensis* (*N* = 10). The heatmap scale ranges from − 2 to + 2 on a *log*_*2*_ scale. **b** KEGG analysis of all the identified metabolites. **c** HMDB Super Class analysis of all the identified metabolites

The untargeted metabolomic analysis identified 3387 metabolites with annotations (Additional file 1). Three quality checking parameters, including total ion chromatograms, *m/z* widths and retention time widths, were tested, indicating that the data generated a high degree of overlap and the UPLC-MS/MS analysis reached the required standards (Additional file 2). A PCA showed that the percentages of the explained values of PC1 and PC2 were 42.12 and 18.63%, respectively, indicating a clear separation of the metabolomes from *P. chienii* and *T. yunnanensis* (Additional file 3). The metabolite profiling of the two Taxaceae species revealed great variations in their metabolomes (Fig. 2a).

According to their annotations, many metabolites were assigned into different metabolic pathways. KEGG enrichment analysis showed that most metabolites were belonged to amino acid metabolism, carbohydrate metabolism, terpenoids and polyketides metabolism, and cofactors and vitamins metabolism (Fig. 2b). HMDB Super Class analysis showed that 311 metabolites were grouped to 11 major categories, such as “organic acids and derivatives” (161 metabolites), “organoheterocyclic compounds” (31 metabolites), “organooxygen compounds” (24 metabolites), “phenylpropanoids and polyketides” (23 metabolites), and “nucleosides, nucleotides, and analogue” (17 metabolites) (Fig. 2c).

### Overview of the transcriptome

Using similar samples, RNA-sequencing yielded 50.35 Gb of sequence data, including 25.40 Gb from *P. chienii* and 24.95 Gb from *T. yunnanensis* (Additional file 4). The clean reads were assembled and produced 133,507 transcripts (N50: 1561), with a mean length of 513 bp, and 61,146 unigenes (N50: 1606), with a mean length of 419 bp (Additional file 5). Analysis of size distribution of all transcripts showed that 11.48% of the transcripts 11.01% of the unigenes were > 2000 bp in length (Additional file 5). For annoatation, 61,146 unigenes were annotated by several common databases (Additional file 5). The species distribution suggested that the majority of the unigenes displayed significant similarities to known proteins from *Picea sitchensis*, *Amborella trichopoda*, and *Quercus suber* (Additional file 5).

**Fig. 3:**
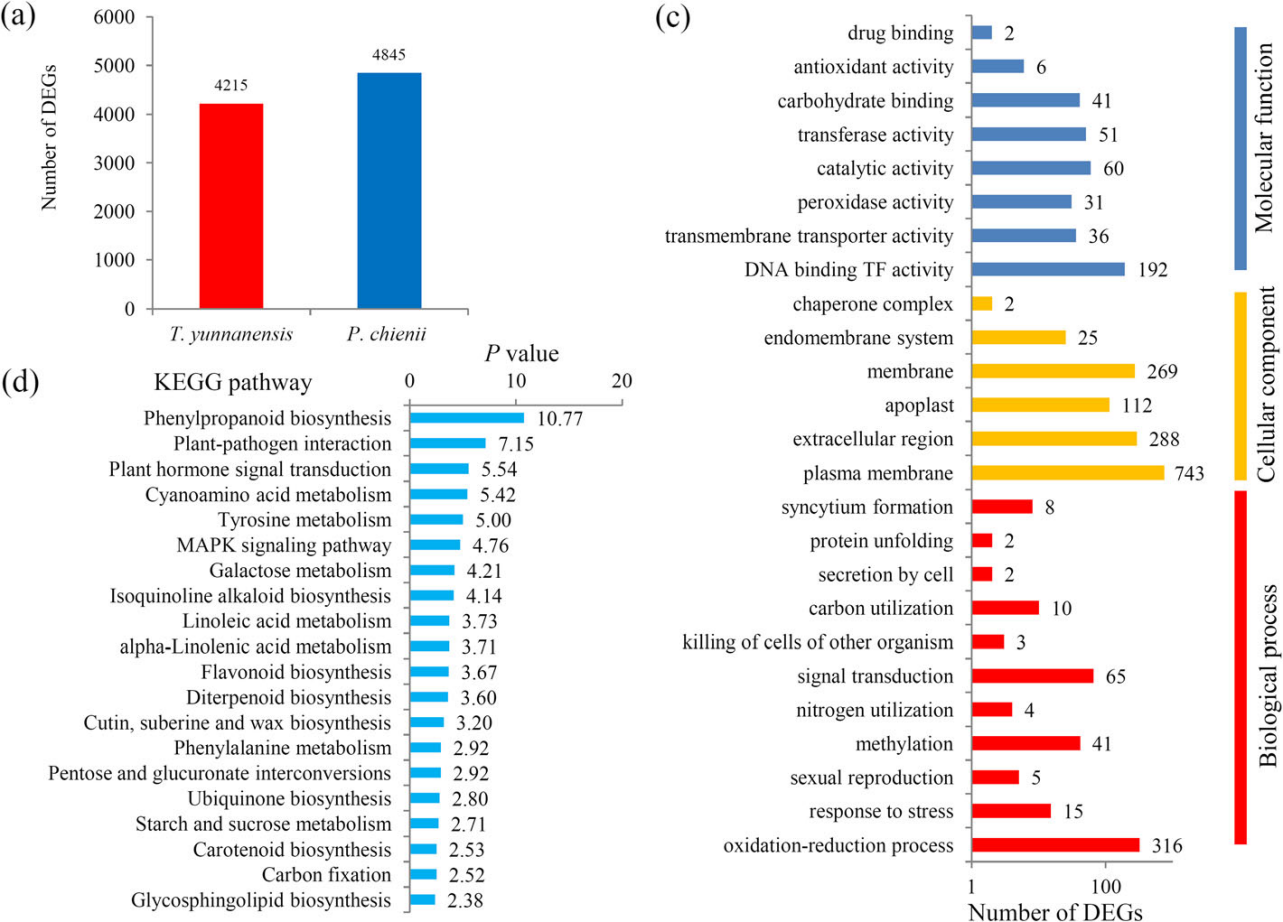
Identification of the DEGs between *P. chienii* and *T. yunnanensis*. **a** The numbers of *P. chienii* predominantly expressed genes and *T. yunnanensis* predominantly expressed genes. **b** GO analysis of all the DEGs between *P. chienii* and *T. yunnanensis*. **c** KEGG analysis of all the DEGs between *P. chienii* and *T. yunnanensis*

Analysis of DEGs showed 4,215 *T. yunnanensis* highly-expressed unigenes and 4,845 *P. chienii* highly-expressed unigenes (Fig. 3a). Most of the DEGs were assigned into different GO terms belonging to three major categories (Fig. 3b and Additional file 6). KEGG analysis showed that 34 KEGG pathways were significantly enriched in the DEGs between *T. yunnanensis* and *P. chienii* (Additional file 7). The top 20 enriched KEGG pathways, such as the ‘phenylpropanoid biosynthesis’, ‘plant-pathogen interaction’, and ‘plant hormone signal transduction’ pathways, were shown (Fig. 3c).

### Variations in primary and secondary metabolism between *T. yunnanensis* and *P. chienii*

**Fig. 4:**
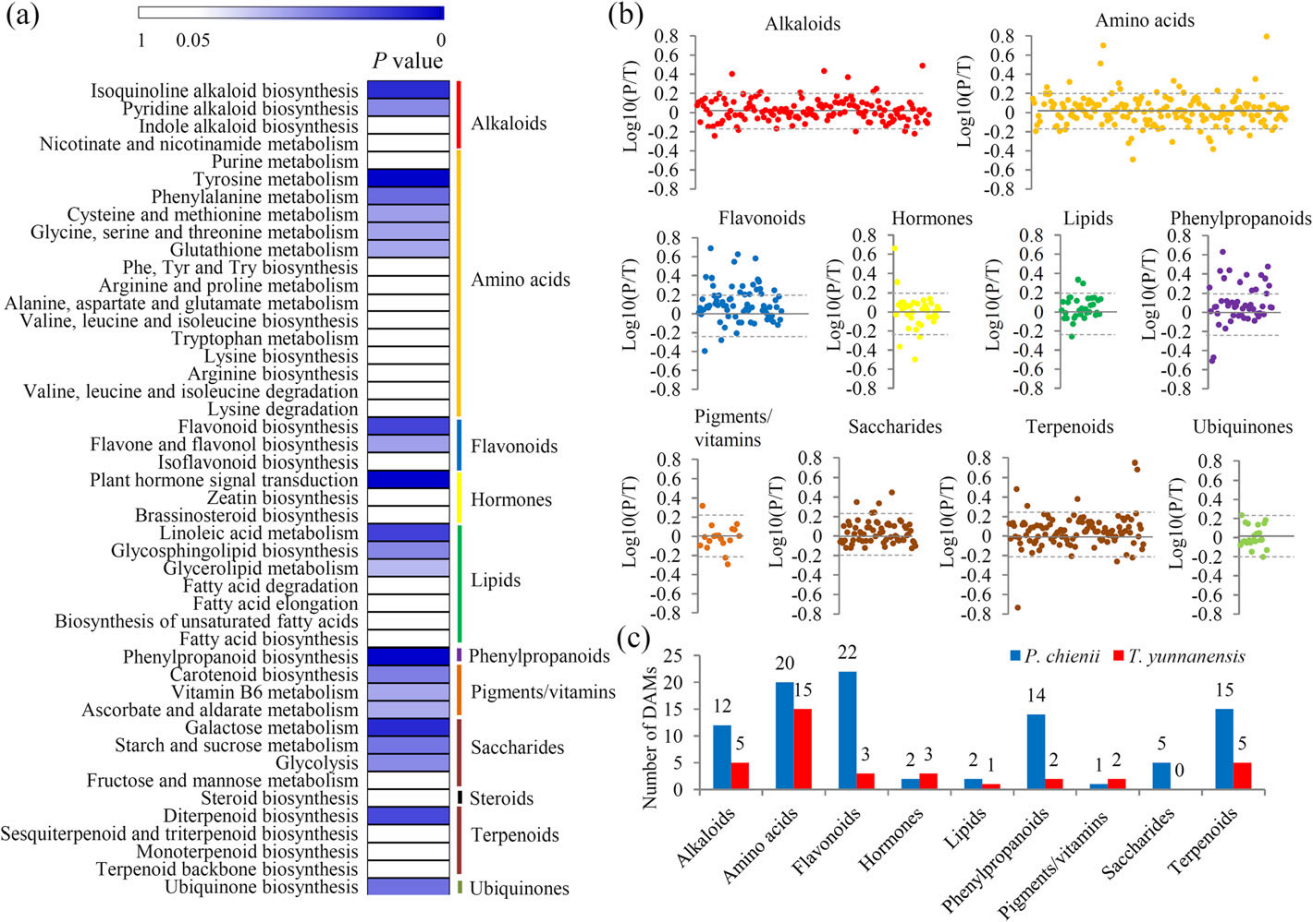
Comparative analysis of DEGs and DAMs between *P. chienii* and *T. yunnanensis*. **a** KEGG enrichment analysis of the DEGs. The significant *P* value of each KEGG term between *P. chienii* and *T. yunnanensis* was shown by a heatmap. All the KEGG terms were grouped into 11 metabolism-related categories, which were indicated by different color bars. **b** The relative abundances of the metabolites belonging to various major metabolic categories. **c** The numbers of *P. chienii* predominantly accumulated and *T. yunnanensis* predominantly accumulated metabolites in different metabolic categories

According to their annotations, a large number of DEGs were involved in the primay and secondary metabolism, and the majority of the DEGs were grouped into 46 KEGG terms belonging to 11 major categories. Significance values of each KEGG term were calculated and shown in Additional file 8. In detail, two alkaloid-related pathways, five amino acid-related pathways, two flavonoid-related pathways, one hormone-related pathway, three lipid-related pathways, one phenylpropanoid-related pathway, all three pigment/vitamin pathways, three saccharide-related pathways, one terpenoid-related pathway, and one ubiquinone-related pathway, showed significant differences between *T. yunnanensis* and *P. chienii* (Fig. 4a).

Untargeted metabolomic analysis identified 313 differentially accumulated metabolites (DAMs), 129 DAMs of which were assigned into different primary and secondary metabolite categories (Fig. 4b). The numbers of DAMs belonging to each category were shown in Fig. 4c.

### Variations in the precursors of taxol biosynthesis between *T. yunnanensis* and *P. chienii*

**Fig. 5:**
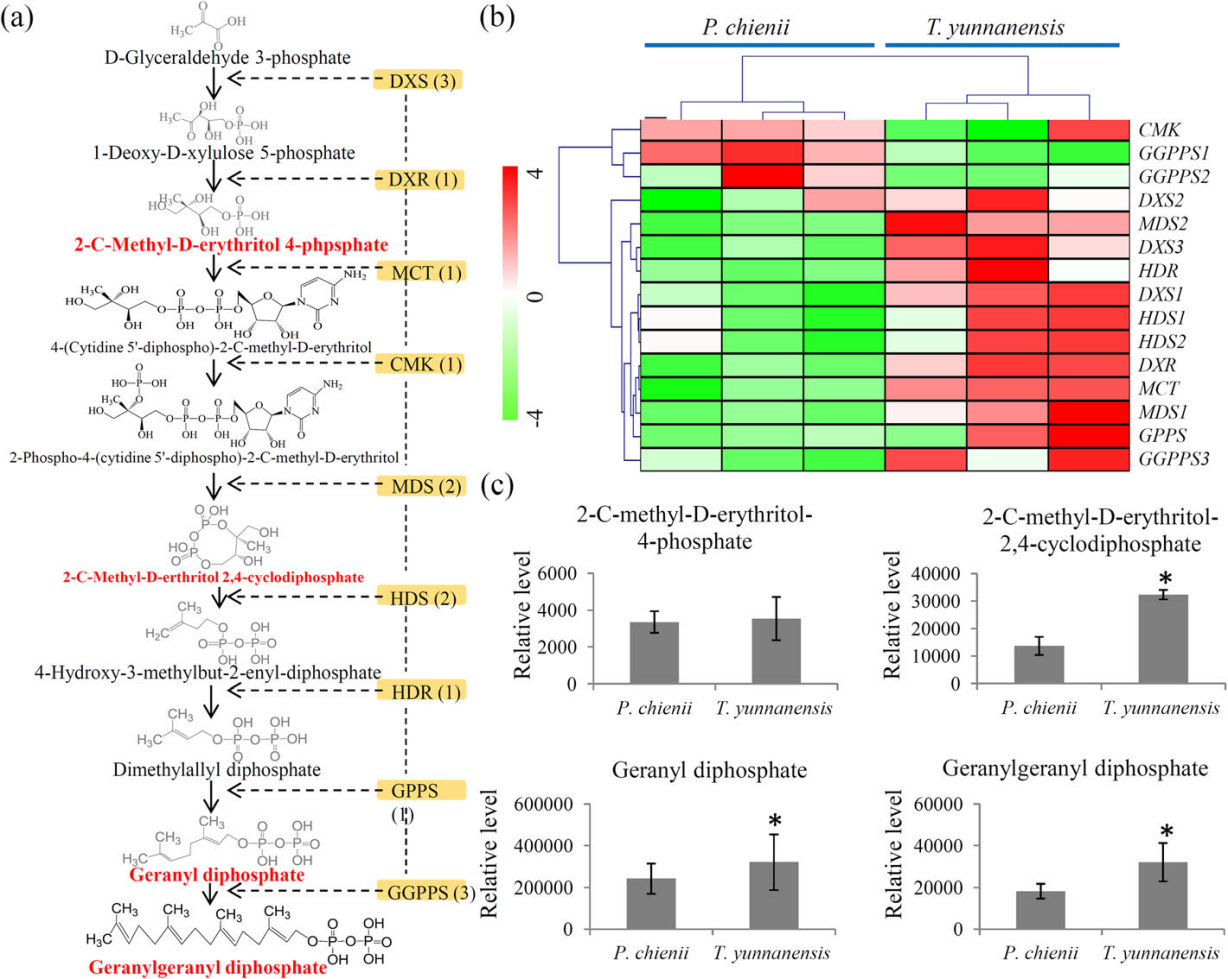
Integrated metabolomic and transcriptomic analysis of the MEP pathway. **a** Overview of the MEP pathway. The orange backgrounds indicated the genes identified by the transcriptome and red font indicated the metabolites identified by the metabolome. **b** Differential expression of the key genes involved in the MEP pathway. The heatmap scale ranges from − 4 to + 4 on a *log*_*2*_ scale. **c** Differential accumulation of the intermediate metabolites involved in the MEP pathway. “*” represents significant differences (*P* < 0.05)

The MEP pathway provided a key precursor, GGPP, for taxol biosynthesis [35]. Based on the sequence similarity to model plants, a predicted MEP pathway is showed in Fig. 5a. Our transcriptome data revealed at least one unigene encoding one enzyme that is involved in the MEP pathway (Additional file 9). In the MEP pathway, three DXS encoding unigenes, one DXR encoding unigene, one MCT encoding unigene, one CMK encoding unigene, two MDS encoding unigene, two HDS encoding unigenes, one HDR encoding unigene, one GGPS encoding unigene, and three GGPPS encoding unigenes, were identified. Most of the MEP pathway-related genes highly expressed in *T. yunanensis*, except for *CMK*, *GGPPS1* and *GGPPS2* (Fig. 5b).

Furthermore, our metabolome data identified four intermediate metabolites of the MEP pathway, including 2-C-methyl-D-erythritol 4-phosphate, 2-C-methyl-D-eryhritol 2,4-cyclodiphosphate, geranyl diphosphate (GPP), and GGPP. Among these intermediate products, 2-C-methyl-D-eryhritol 2,4-cyclodiphosphate, GPP, and GGPP highly accumulated in *T. yunnanensis* (Fig. 5c).

### Variations in the taxol biosynthesis pathway between *T. yunnanensis* and *P. chienii*

**Fig. 6:**
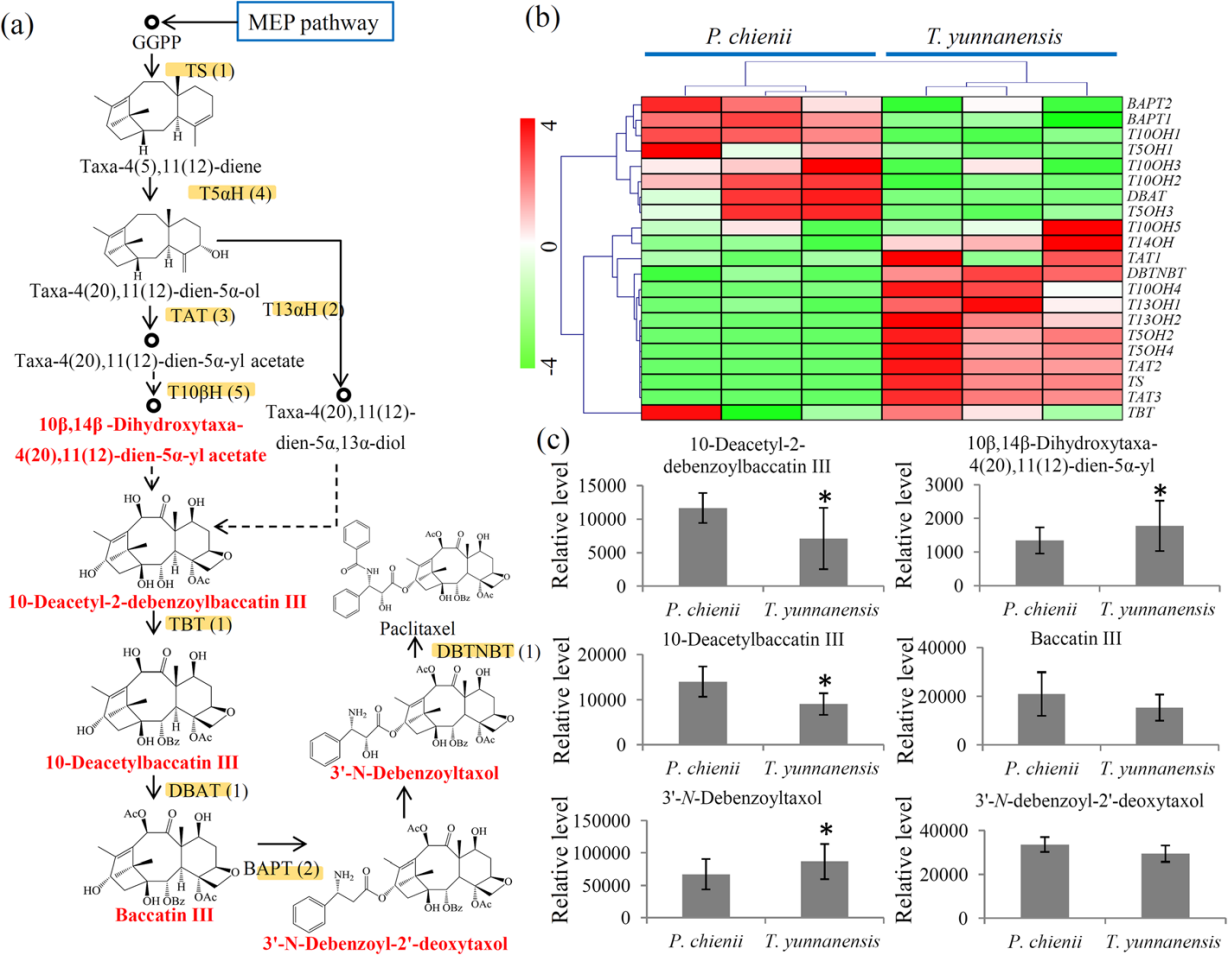
Integrated metabolomic and transcriptomic analysis of the taxol biosynthesis pathway. **a** Overview of the taxol biosynthesis pathway. The orange backgrounds indicated the genes identified by the transcriptome and the red fonts indicated the metabolites identified by the metabolome. **b** Differential expression of the key genes involved in the taxol biosynthesis pathway. The heatmap scale ranges from − 4 to + 4 on a *log*_*2*_ scale. **c** Differential accumulation of the intermediate metabolites involved in the taxol biosynthesis pathway. “*” represents significant differences (*P* < 0.05)

Taxol biosynthesis involves a complicated metabolic pathway consisting of several intermediate products and their catalyzing enzymes [9]. In our study, 20 unigenes encoding nine key enzymes involved in the taxol biosynthesis pathway were identified, including one TS encoding gene, four T5αH encoding genes, three TAT encoding genes, two T13αH encoding genes, five T10βH encoding genes, one TBT encoding gene, one DBTNBT encoding gene, one DBAT encoding gene and two BAPT encoding genes (Fig. 6a and Additional file 10). The *BAPT1/2*, *DBAT*, *T5αH1/3*, *T10βH1/2/3* genes highly expressed in *P. chienii* and the *TAT1/2/3*, *DBTNBT*, *T10βH4*, *T13*α*H1/2*, *TBT* and *TS* genes greatly expressed in *T. yunnanensis* (Fig. 6b).

Our metabolome data identified six intermediate metabolites involved in taxol biosynthesis. Among these intermediate metabolites, 10-deacetyl-2-debenzoylbaccatin, and 10-deacetylbaccatin highly accumulated in *P. chienii* and 10β,14β-dihydroxytaxa-4(20),11(12)-dien-5α-yl acetate and 3′-*N*-debenzoyltaxol highly accumulated in *T. yunnanensis* (Fig. 6c).

### Variations in dead-end metabolites of taxol biosynthesis and 14-hydroxylated taxoids between *T. yunnanensis* and *P. chienii*

**Fig. 7:**
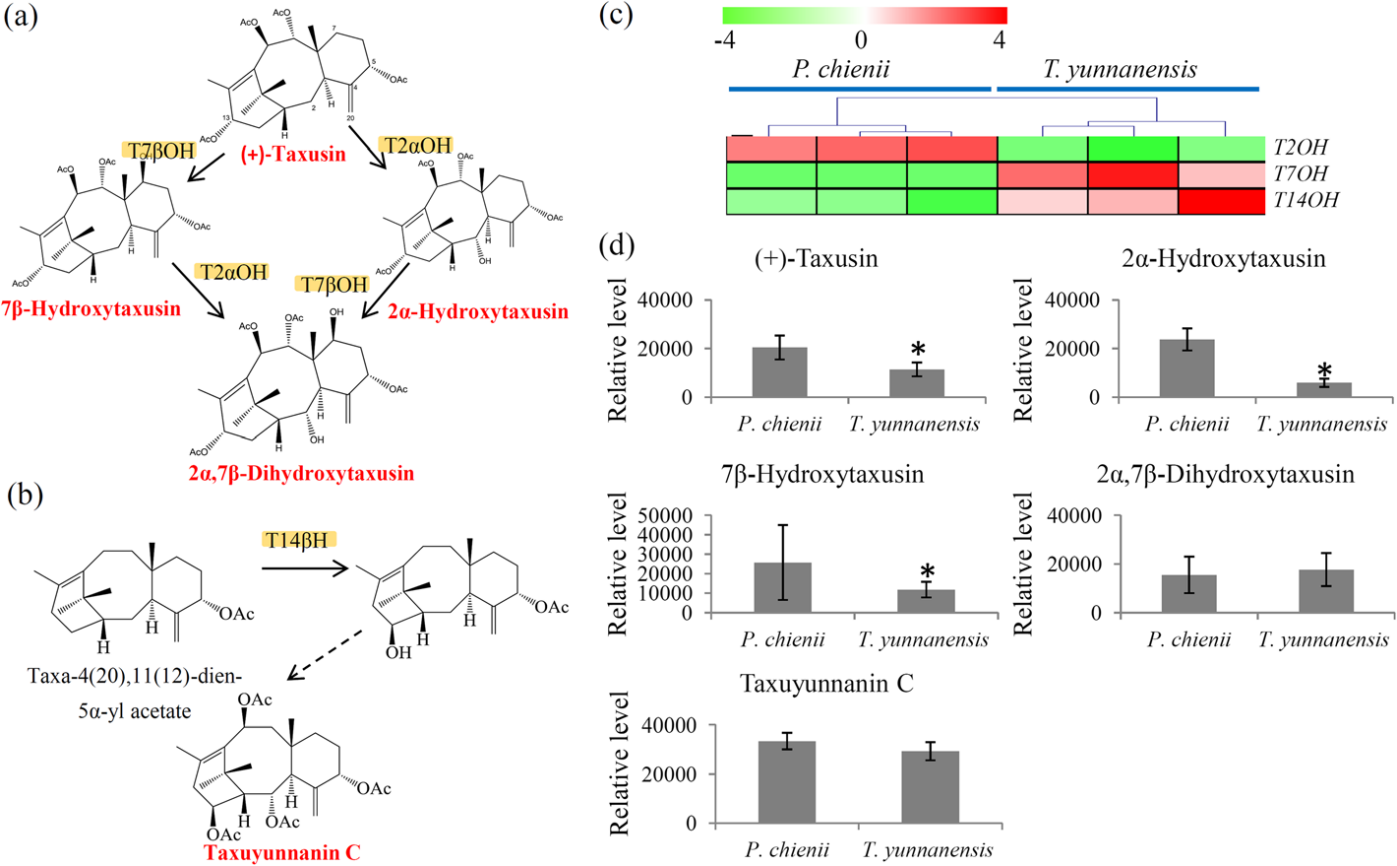
Integrated metabolic and transcriptomic analysis of the branch of taxol biosynthesis pathway. The overview of the taxusin metabolism (**a**) and taxuyunnanin C metabolism (**b**) pathway. Orange background indicated the genes identified by the transcriptome and red font indicated the metabolites identified by the metabolome. (**c**) Differential expressed key genes involved in the branch of taxol biosynthesis pathway. The heatmap scale ranges from -4 to +4 on a log_2_ scale. (**d**) Differential accumulation of the metabolites involved in the branch of taxol biosynthesis pathway. “*” represents significant differences (*P* < 0.05)

Our transcriptome identified the encoding genes of T2αH and T7βH that are involved in the metabolism of taxusin-like metabolites and the encoding gene of T14βH that is involved in the biosynthesis of taxuyunnanin C, a classic 14-hydroxylated taxoid (Fig. 7a and b). The *T2αH* gene highly expressed in *P. chienii*, while *T7βH* and *T14βH* genes predominantly expressed in *T. yunnanensis* (Fig. 7c). Furthermore, several dead-end metabolites, such as (+)-taxusin, 2α-hydroxytaxusin, 7β-hydroxytaxusin and 2α, 7β-dihydroxytaxusin, and one 14-hydroxylated taxoid, taxuyunnanin C, were identified by the metabolomic analysis. The results showed that (+)-taxusin, 2α-hydroxytaxusin, and 7β-hydroxytaxusin highly accumulated in *P. chienii*. No significant differences in the levels of 2α, 7β-dihydroxytaxusin and taxuyunnanin C between *P. chienii* and *T. yunnanensis* were obersved (Fig. 7d).

### Expression validation of the key genes involved in the taxol pathway

**Fig. 8:**
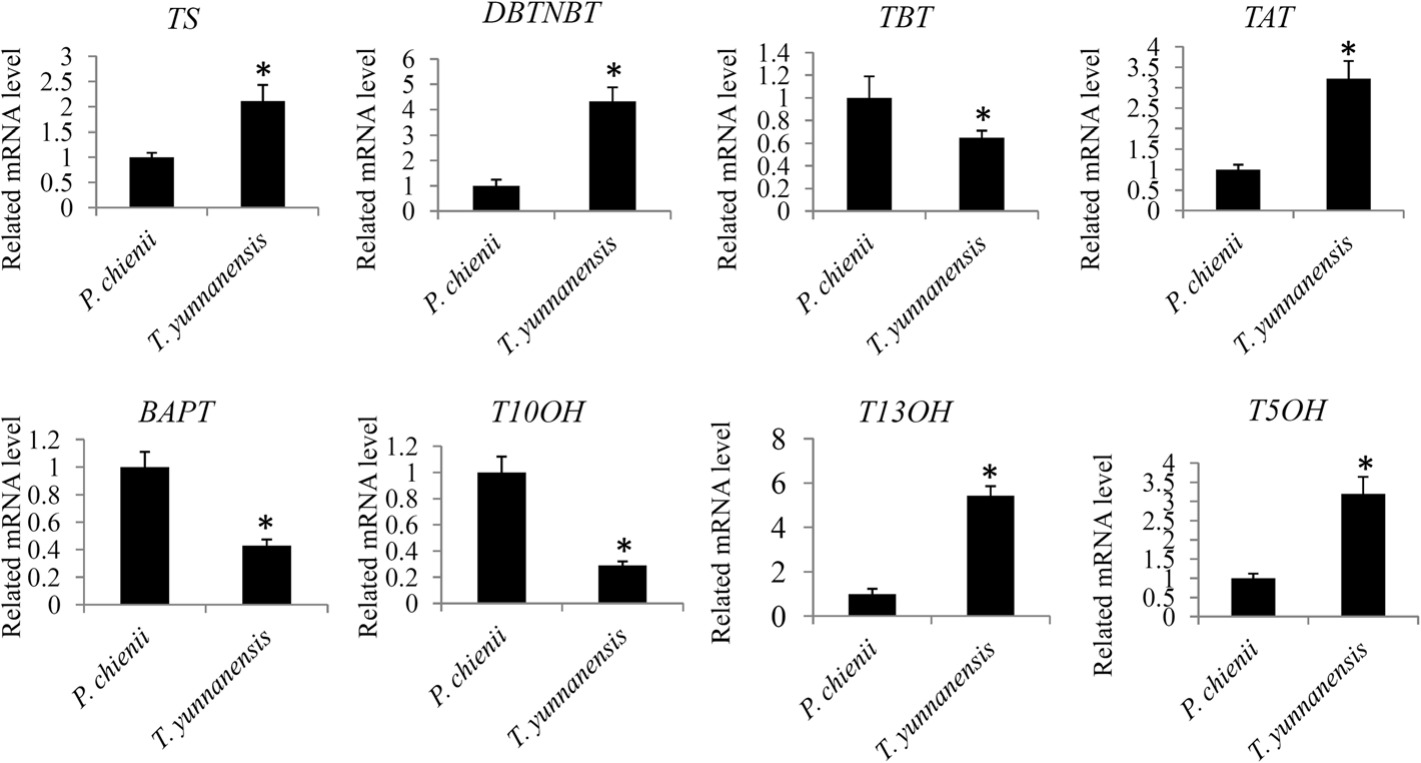
Expression validation of the key genes involved in the taxol pathway. The significant variations (*P* < 0.05) are indicated by ‘*’ and error bars represent mean ± SD (*N* = 3)

To investigate the differences in expression levels of the key genes involved in the taxol pathway, the relative levels of eight randomly selected taxol pathway-related genes were determined by qRT-PCR analysis. *TS*, *DBTNBT*, *TAT*, *T13OH*, *T5OH* genes were highly expressing in *T. yunnanensis* and *TBT*, *BAPT*, *T10OH* genes highly expressing in *P. chienii* (Fig. 8).

## Discussion

To date, *Taxus* plants are the sole natural resource for taxol extraction, limiting the supplyment of taxol [36]. Huge economic values of taxol make the selection of high taxol-yielding Taxaceae species a topical research area [2, 32, 33]. Our data confirmed the occurence of taxol and other taxoids in *P. chienii* for the first time, suggesting that taxol is not the exclusive metabolite of *Taxus* genus. Further analysis showed that *P. chienii* contains a higher level of taxol than *T. yunnanensis*, indicating *P. chienii* a good taxol-yielding species for large-scale cultivation. Taking *T. yunnanensis* as a representative *Taxus* tree, an integrated metabolomic and transcriptomic approach was employed to elucidate the species-specific accumulations of taxoids between *P. chienii* and *Taxus* trees.

As the first transcriptome of *P. chienii*, our data provided 61,146 unigenes with full length or partial sequences, which is an important foundation for the investigation of the taxol biosynthesis pathway in *P. chienii*. Based on the unigene pool, we predicted the MEP pathway in *P. chienii*, which supplies a key precursor for the diterpenoid taxane core [35] (Fig. 5a). According to the metabolomes, GGPP was detected in both *P. chienii* and *T. yunnanensis*. In addition, an outline of taxol biosynthesis pathway, including 20 unigenes encoding nine key enyzmes, was also predicted (Fig. 6a). In the taxol biosynthesis pathway, six essential intermediates were identified in *P. chienii*. Our data suggested the presence of a classic taxol biosynthesis pathway in *P. chienii*.

The MEP pathway supplies three units of IPP and one unit of DMAPP to synthesize the diterpenoid taxane core [37]. The up-regulation of the MEP pathway was considered to have a positive effect on the precursor supply [38]. For example, DXS and DXR have been implicated as catalyzing slow steps in the biosynthesis of plastid-derived terpenoids [39]. GGPPS is another important enzyme that plays a regulatory role in taxoid production [40, 41]. Compared to *T. yunnanensis*, the expression levels of three *DXS* genes, one *DXR* gene and one *GGPPS* gene were relatively lower in *P. chienii* (Fig. 5b). The metabolomes confirmed that two major terminal products of the MEP pathway, GPP and GGPP, highly accumulated in *T. yunnanensis*, suggesting a more abundant precursor supply in *T. yunnanensis*.

In addition to taxol, several important imtermediate products of taxol biosynthesis also showed differential accumulations between *P. chienii* and *T. yunnanensis*. TS, catalyzing the formation of the intermediate product taxa-4(5),11(12)-diene, is a rate-limiting enzyme in the taxol biosynthesis pathway [18, 42]. TAT catalyzes an important step of taxol biosynthesis, which is responsible for the acetylation of taxa-4(20),11(12)-dien-5α-ol [43]. In our study, one unigene encoding TS and three unigenes encoding TAT were identified, and they predominantly expressed in *T. yunnanensis*. DBAT, another rate-limiting enzyme, catalyzes the formation of baccatin III from 10-deacetylbaccatin III [44]. BAPT is responsible for the transfer of a C13-side chain to baccatin III [45]. Here, one unigene encoding DBAT and two unigenes encoding BAPT were identified, and they predominantly expressed in *P. chienii*. In pharmaceuticals industry, baccatin III and 10-DAB are key starting material of pacitaxel [46]. Our data showed that baccatin III and 10-DAB highly accumulated in *P. chienii*, suggesting a great economic value of *P. chienii* extracts. The differential expression levels of these key genes provided a possible explanation for the differential accumulation of the intermatdite products of taxol biosynthesis between *T. yunnanensis* and *P. chienii*.

The presence of dead-end metabolites and 14-hydroxylated taxoids in *Taxaceae* trees has been previously reported [17]. For example, (+)-taxusin, a dead-end metabolite divergent from taxol formation, is a prominent metabolite of yew heartwood [47]. Taxusin-like metabolites display great anti-inflammatory and antinociceptive activities and are alternate substrates for testing microsomal oxygenase activities [47]. Interestingly, (+)-taxusin and its hydroxylated products highly accumulated in *P. chienii*, suggesting a wider value of *P. chienii* in pharmaceutical industry.

Recently, a large number of TFs have been considered to be involved in the regulation of taxol biosynthesis [48]. For examples, TcJAMYC1/2/3, TcERF12/15, and TcWRKY1/8/47 regulate the expression of paclitaxel biosynthetic genes by binding to their promoters [49–51]. In our study, 12 differentially expressed bHLH genes, 24 differentially expressed ERF genes, and 16 differentially expressed WRKY genes were identified, providing more candidate regulators for the taxol biosynthesis. The MYC TFs are key regulators of the jasmonic acid (JA) signaling pathway and MeJA can improve the production of taxol [52]. In *T. media*, a number of biosynthesis gene promoters could be activated by MYC2, MYC3 and MYC4 [53]. In our study, 3 of 4 MYC TFs showed differential expressions, indicating potential differences in MYC-mediated JA signaling transduction between *P. chienii* and *T. yunnanensis* [54]. Differenially expressed MYC TFs might play an important role in taxol biosynthesis. Interestingly, a phloem-specific TmMYB3 was reported to be involved in the transcriptional regulation of paclitaxel biosynthesis [55]. Recently, expression profiling and posttranscriptional regulation of the R2R3-MYB TF family in *T. chinensis* have been well analyzed [56]. In our study, a number of MYB genes were identified, suggesting potential tissue-specific accumulation of taxoids in both *P. chienii* and *T. yunnanensis*.

## Conclusions

In summary, the occurrence of taxol and other taxoids in *P. chienii* was confirmed by a UPLC-MS/MS method. Several taxoids, such as 10-DAB, taxol, and 7-E-PTX, highly accumulated in *P. chienii*, suggesting that it is a good taxol-yielding species for large-scale cultivation. Comparative metabolomic and transcriptomic analyses revealed the variations in the precursors, intermediate products and dead-end metabolites of taxol biosynthesis between *P. chienii* and *T. yunnanensis*. Furthermore, a number of differentially expressed TFs between *P. chienii* and *T. yunnanensis* were also identified, providing novel candidate regulators of taxol biosynthesis. Our study may aid in better understanding of the differences between *Pseudotaxus* and *Taxus* trees, and promoting comprehensive resource utilization of *P. chienii*, an endangered and rarely studied Taxaceae tree.

## Methods

### Plant materials and sampling

Cultivated 5-year-old *P. chienii* and *T. yunnanensis* trees were planted in a greenhouse at the campus of Hangzhou Normal University, Hangzhou, China, with a light/dark cycle of 12/12 h and 60% ~ 70% relative humidity at temperature of 25 ± 1 °C. The authorities responsible for the *Taxus* resources are the Mount Tianmu National Nature Reserve, who provided permission to collect the samples of *P. chienii*, and Motuo National Nature Reserve, who provided permission to collect the samples of *T. yunnanensis*. The formal identification of the plant material was undertaken by Dr. Lei Zhang (Washington State University). A voucher specimen of this material has not been deposited in a publicly available herbarium. Experimental researches on *Tauxs* trees comply with Hangzhou Normal university guidelines. For metabolomic analysis, fresh twigs were harvested from 10 independent trees of *P. chienii* and *T. yunnanensis*, respectively. For transcriptomic analysis, fresh twigs were harvested from 3 independent trees of *P. chienii* and *T. yunnanensis*, respectively. All the samples were ground in liquid nitrogen and transferred to a tube.

### Metabolite extraction and sample preparation

Each sample was added mixed with an aliquot of 500 μL pre-colded 50% methanol with several steel balls. The mixture solution was shaken at a rate of 1900 strokes/min for 2 min using a 2010 Geno/Grinder (SPEX SamplePrep, Metuchen, NJ, USA). After centrifugation at 4000 g for 20 min, the supernatants were transferred into new 96-well plates. The samples were stored at − 80 °C prior to the UPLC-MS/MS analysis. In addition, the quality control samples were also prepared by combining 10 μL of each extraction mixture.

### Untargeted metabolomic profiling

All samples were analyzed by the UPLC-MS/MS system according to our previous work [32]. Firstly, all chromatographic separations were performed using an ultra performance liquid chromatography (UPLC) system (SCIEX, UK). An ACQUITY UPLC BEH Amide column (100 mm × 2.1 mm, 1.7 μm, Waters, Milford, MA, USA) was used for the reversed phase separation. The column oven was maintained at 35 °C. The flow rate was 0.4 mL/min and the mobile phase consisted of solvent A (25 mM ammonium acetate and 25 mM NH_4_H_2_O) and solvent B (IPA:CAN = 9:1, v/v, and 0.1% of formic acid). Gradient elution conditions were set as follows: 0 ~ 0.5 min, 95% of solvent B; 0.5 ~ 9.5 min, 95 to 65% of solvent B; 9.5 ~ 10.5 min, 65% ~ 40% of solvent B; 10.5 ~ 12 min, 40% of solvent B; 12 ~ 12.2 min, 40% ~ 95% of solvent B; 12.2 ~ 15 min, 95% of solvent B. The injection volume for each sample was set at 4 μL.

A high-resolution MS/MS TripleTOF 5600 plus (SCIEX, UK) was used to recognize the metabolites eluted from the column. The TOF was carried out in both positive and negative ion modes. The detail parameters of UPLC-MS/MS analysis were set according to our previous work [32]. Furthermore, in order to evaluate the stability of the UPLC-MS/MS system during the whole data acquisition process, one quality control sample was detected after every 10 samples.

### Bioinformatics of the metabolomic datasets

The acquired MS data features, including peak picking, peak grouping, retention time (RT), second peak grouping, and annotation of isotopes and adducts was performed using XCMS software [57]. Each ion was identified by combining RT and m/z data together. Intensities of each peak were recorded and a 3D matrix, containing each assigned peak indexs (retention time-m/z pair), sample name and ion intensity, was generated.

Metabolites were annotated by matching the exact m/z of samples with those from the online KEGG and PLANTCYC database. The molecular formulas of all annoated metabolites were further validated by the isotopic distribution measurements. The intensity of peak data was further preprocessed by an in-house software metaX. Low quality features that were detected in less than 50% of quality control samples or 80% of experiment samples were removed. The high quatily peaks were imputed with the k-nearest neighbor algorithm to further improve their quality. PCA was performed for outlier detection and batch effects evaluation using the pre-processed dataset. In addition, the relative standard deviations (SD) of the metabolic features were calculated across all quality control samples to remove the features with SD > 30%.

To screen differential accumulated metabolites, the default setting parameters, such as VIP > 1, *P* < 0.05, were used. The statistical analyses in metabolomics, including univariate analysis and multivariate PLS-DA analysis with corresponding VI*P* value, were performed. The univariate analysis is Wilcoxon test with *P* value corrected by BH correction.

### RNA extraction and sequencing

Total RNAs were extracted using an RNeasy plant mini kit (Qiagen, Hilden, Germany) according to its manual. Then, 10 μg of each RNA sample (three biological replicates) was used for library construction. cDNA library construction was performed according to the method previously described by Yu et al. [30]. Sequencing was performed using an Illumina Hiseq 4000 platform (LC-Bio, Hangzhou, China) according to its protocol.

Raw RNA-seq reads were trimmed for low quality reads with length shorter than 25 bp. Based on the clean reads, transcriptomic assembly was perfromed using Trinity software [58]. The raw sequence data have been submitted to the NCBI Short Read Archive with accession number GSE121523 and GSE121831.

### Analysis of the transcriptomic datasets

For gene annotation, all assembled sequences were searched against various databases, such as non-redundant (Nr) protein, Gene Ontology (GO), SwissProt, and Kyoto Encyclopedia of Genes and Genomes (KEGG) databases. We used the blastx function in dismond software to search the assembled genome against with different databases, setting evalue < 0.00001. When a gene sequence could be aligned to multiple protein, the result with minimun evalue were selected as the final annotation of the gene. According to the protein ID, the conrresponding gene symbol is obtained.

Expression levels for the unigenes were calculated by transcripts per million method. The transcript abundance values of each unigene in different sample groups were transformed into Z-score after log transformation. The differentially expressed genes (DEGs) were screened with criterions: log2(fold change of transcript abundance) > 1 and statistical significance *P* < 0.05. GO and KEGG enrichment analysis of the DEGs were performed on the DEGs by perl scripts in house. The heatmap was made using MultiExperiment Viewer (version 4.9.0). Software samtools was used to analyze and filter the SNP with default parameters. All SNPs with mindepth less than 100 were removed.

### Quantificative analysis of targeted taxoids

Fresh twigs were collected from six independent trees of each species. The samples were thoroughly dried at 40 °C and then ground into fine powder. A modified version of a previously published method was used to prepare crude extracts [30]. Six common taxoids, including 10-deacetylbaccatin III (10-DAB), baccatin III (BAC), 10-deacetylpaclitaxel (DAP), paclitaxel (PTX), 7-epi 10-desacetyl paclitaxel (7-E-DAP) and 7-epipaclitaxel (7-E-PTX) were quantified using a UPLC-MS/MS method. Before UPLC-MS/MS analysis, the crude extracts were diluted at a ratio of 1:10 and were passed through 0.22 μm membrane filters.

For the separation of these taxoids, a Kinetex C_18_ column (100 × 4.6 mm, 2.6 μm, Phenomenex, Torrance, CA, USA) was used. The determination of these taxoids was performed using a LC-30 AD UPLC (Shimadzu, Japan) coupled with a SCIEX QTRAP 6500 MS (Applied Biosystems). The Multi-Quant software (version 3.0) was applied for data acquisition and processing. Multiple reaction monitoring (MRM) was used in the positive ionization mode. The transitions of *m/z* 567.4 → 445.3 for 10-DAB, *m/z* 609.5 → 427.3 for BAC, *m/z* 834.4 → 308.2 for DAP, *m/z* 876.4 → 308.2 for PTX, *m/z* 876.4 → 591.4 for 7-E-PTX, and *m/z* 834.4 → 308.2 for 7-E-DAP were used for the quantification. The quantification of seven taxoids was carried out using an LC-30 AD UPLC system (Shimadzu, Japan) coupled with a SCIEX QTRAP 6500 mass spectrometer (Applied Biosystems). The results were presented as the means of at least three replicates ± standard errors.

### Real-time PCR validation

Real-time PCR experiment was performed according to our previous work [30]. In brief, the SYBR Premix Ex Taq Kit (TaKaRa, Dalian, China) and a DNA Sequence Detection System (ABI PRIM 7700) were used. Independent cDNA samples from *P. chienii* and *T. yunnanensis* were used for real-time PCR experiments. An ACTIN sequence was used as the internal standard gene to calculate relative fold differences by the values of comparative cycle threshold (2^−ΔΔCt^). The expression analysis was performed for three biological replications. The primer sequences were listed in Additional file 12.

### Statistical analysis

Statistical analyses were carried out using SPSS software version 19.0 (SPSS Inc., Chicago, IL, USA), and a one-way ANOVA was applied to compare the differences in taxoid contents between the two sample groups. Wilcoxon tests were conducted to detect differences in metabolite concentrations between two species. The *P* value was adjusted for multiple tests using an FDR (Benjamini–Hochberg). Supervised PLS-DA was conducted through metaX to discriminate the different variables between two groups. The VIP value was calculated. A VIP cut-off value of 1.0 was used to select important features.

## Supplementary Information

**Additional file 1: Table S1.** The detailed information of 3387 metabolites with annotations.

**Additional file 2: Figure S1.** Quality control parameters of the metabolomes.

**Additional file 3: Figure S2.** PC analysis of the metabolomes of *P. chienii* and *T. yunnanensis*.

**Additional file 4: Table S2.** Detailed information of the transcriptomes.

**Additional file 5: Figure S3.** Length distribution of assembled transcripts and unigenes.

**Additional file 6: Table S3.** GO categories of all the DEGs between *T. yunnanensis* and *P. chienii*.

**Additional file 7: Table S4.** KEGG enriched analysis of the DEGs between *T. yunnanensis* and *P. chienii*.

**Additional file 8: Table S5.** Significance values of each KEGG term belonging to primary and secondary metabolism.

**Additional file 9: Table S6.** The detailed information of the unigenes encoding the enzymes that are involved in the MEP pathway.

**Additional file 10: Table S7.** The detailed information of the unigenes encoding the enzymes that are involved in the taxol biosynthesis pathway.

**Additional file 11: Table S8.** The number of identified TFs and differentially expressed TFs.

**Additional file 12: Table S9.** The primer sequences for qRT-PCR.

## Data Availability

The raw sequence data has been submitted to the NCBI Short Read Archive with accession numbers GSE121523 and GSE121831.

## Abbreviations

GGPP: Geranylgeranyl diphosphate
IPP: Isopentenyl diphosphate
DMAPP: Dimethylallyl diphosphate
MEP: 2-C-methyl-D-erythritol phosphate
TS: Taxadiene synthase
10-DAB: 10-deacetylbaccatin-III
10-DAB III: 10-deacetylbaccatin III
HPLC-MS/MS: High-performance liquid chromatography-tandem mass spectrometry
ANOVA: Analysis of variance
PCA: Principal component analysis
DAM: Differential accumulated metabolite
