# Supplementary material for: Omic analysis of the endangered Taxaceae species Pseudotaxus chienii revealed the differences in taxol biosynthesis pathway between Pseudotaxus and Taxus yunnanensis trees
Source: BMC Plant Biol. 2021 Feb 19;21:104. doi: 10.1186/s12870-021-02883-0 (PMC7903646; doi:10.1186/s12870-021-02883-0)
Supplement: Supplementary file 2 — Additional file 2: Figure S1. Quality control parameters of the metabolomes. [file 12870_2021_2883_MOESM2_ESM.docx]

Figure S1 **Quality control parameters of the metabolomes.** (a) The total ion chromatograms of all the samples. (b) *m/z* widths and (c) retention-time widths for the metabolomes.
