# Supplementary material for: Omic analysis of the endangered Taxaceae species Pseudotaxus chienii revealed the differences in taxol biosynthesis pathway between Pseudotaxus and Taxus yunnanensis trees
Source: BMC Plant Biol. 2021 Feb 19;21:104. doi: 10.1186/s12870-021-02883-0 (PMC7903646; doi:10.1186/s12870-021-02883-0)
Supplement: Supplementary file 3 — Additional file 3: Figure S2. PC analysis of the metabolomes of P. chienii and T. yunnanensis. [file 12870_2021_2883_MOESM3_ESM.docx]

Figure S2 **PC analysis of the metabolomes of *P. chienii* and *T. yunnanensis*.**
