# Supplementary material for: Omic analysis of the endangered Taxaceae species Pseudotaxus chienii revealed the differences in taxol biosynthesis pathway between Pseudotaxus and Taxus yunnanensis trees
Source: BMC Plant Biol. 2021 Feb 19;21:104. doi: 10.1186/s12870-021-02883-0 (PMC7903646; doi:10.1186/s12870-021-02883-0)
Supplement: Supplementary file 5 — Additional file 5: Figure S3. Length distribution of assembled transcripts and unigenes. [file 12870_2021_2883_MOESM5_ESM.docx]

Figure S3 **Length distribution of assembled transcripts and unigenes.** The detailed information of all clean reads from each sample, which were combined and resulted in 133,507 transcripts (a) and 61,146 unigenes (b). (c) The length distribution of assembled transcripts and unigenes. (d) The number of unigenes annotated by different databases, including GO, KEGG, Pfam, Swiss-Prot, eggNOG and NR. (e) Species distribution of all annotated unigenes.
